# An Optimized Colorimetric Readout Method for Lateral Flow Immunoassays

**DOI:** 10.3390/s18124084

**Published:** 2018-11-22

**Authors:** Jongwon Park

**Affiliations:** Department of Biomedical Engineering, Kyungil University, 50 Gamasil-gil, Hayang-eup, Gyeongsan-si, Gyeongbuk-do 38428, Korea; jpark3@kiu.ac.kr; Tel.: +82-53-600-5721

**Keywords:** colorimetry, photometry, assay reader, quantification

## Abstract

Despite its broad penetration of various markets, the quantitative lateral flow immunoassay (LFIA) suffers from sensitivity issues in some cases. To solve this problem, an optimized colorimetric readout method for LFIA quantification is proposed in this study. An assay reader device utilizing a color camera and an analysis method using a Bayer filtered image were developed. Spectrometric measurements of the assay test line were performed to determine the color channel that contains the test line information and effectively minimizes noise. The change in the intensity ratio with increasing concentration of the target substance in the sample was largest in the green channel. The linear range of the output curve ranged from 0 to 10 ng/mL, and the detection limit was 2 ng/mL. The suggested instrumentation and analysis methods are expected to effectively resolve the low-sensitivity problems of the former LFIA systems and to offer other prospective functionalities for LFIA quantification.

## 1. Introduction

A quantitative lateral flow immunoassay (LFIA) system, consisting of test strips and a reader device, has been broadly used in point-of-care testing for the diagnosis of diseases because of its advantages, such as reasonable sensitivity and specificity as well as rapid time-to-result readouts [1]. LFIA reader devices are classified according to the kind of labeled particles used by the LFIA and the sensor of the reader device. In the case of using a labeled particle that changes in color intensity, a charge-coupled device (CCD) or a complementary metal-oxide-semiconductor (CMOS) camera detection device have been used for assay quantification [2,3]. Additionally, a photodetector with an excitation light source has been applied for the quantification of fluorescent labeled particles in the LFIA [4]. Magnetic labeled particles in the LFIA are magnetized by supplying an external magnetic field and then detected by a magnetic field sensor, such as a giant magnetoresistive sensor [5,6]. Among these, LFIA readers utilizing image sensors, such as a CCD or CMOS, are most commonly used because of the advantages of their simple structure and small size [7]. An image sensor-based LFIA reader acquires an image of the test line, which is the aggregate of the labeled particles, antigens, and antibodies. Then, the pixel intensity of the test line, which changes according to the concentration of the target analyte, is analyzed [8]. However, problems such as the high possibility of false positives and false negatives and limitations for accurate and multiplex quantification have been observed in the utilization of optical readers [1].

The sensitivity of quantitative LFIA systems using optical reader devices is affected by the efficiency of antigen–antibody binding and the colorimetric readout efficiency of traditionally used colloidal gold beads. That is, the sensitivity of the system is determined by the design factors of the test strip and the reader device. Efforts to improve LFIA system sensitivity have mainly focused on changes to the test strip. Research has been conducted on material platforms such as nitrocellulose membranes, colloidal carbon, and fluorescent particles for quantitative or qualitative determination of bound antigen antibodies [1]. In contrast, the degree of improvement in optical reader devices has been relatively stagnant because the development and commercialization of LFIA systems has been dominated by assay developers, and electronic readers are traditionally supplied to them by original equipment manufacturing (OEM) methods without the need for custom performance enhancements [1]. However, the implementation of a fast and accurate point-of-care system using an LFIA should be accompanied by optimization of the associated reader.

LFIA readers utilizing optical sensors quantify labeled particles only in the top 10-μm thick layer of a porous membrane. The remaining signal generated below this thickness does not inherently contribute to particle quantification because the sensor simply cannot detect the signal [9]. Moreover, for all previous approaches utilizing image sensors, monochrome CCD or CMOS cameras have been used for LFIA imaging, in which the overall intensities of all detectable wavelength ranges of the installed monochrome image sensors are taken into consideration to estimate the labeled particle concentration; however, the labeled particle-related color information is only limited to a narrow wavelength range.

This study aimed to improve the performance of an LFIA reader device with an optical sensor. A color image sensor, which had not been used in related reader devices, was implemented in this study, and the obtained color image was filtered with an image of a certain wavelength band [10,11]. In this wavelength range, the signal information from the test line pixel intensity change was included but the noise was minimized. For the quantitative measurement of LFIA, additional optical filters were not required, and an optimized readout method was implemented using red, green, or blue bandpass images through the Bayer filter, already included (i.e., embedded) in the color image sensor.

## 2. Materials and Methods

### 2.1. Spectrometry of Labeled Particles

**Figure 1:**
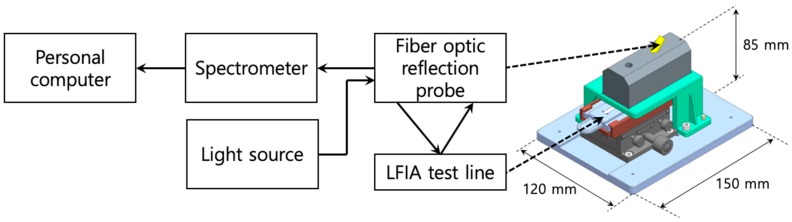
Setup for the spectrometry measurement of the lateral flow immunoassay (LFIA) test line. The white light was from a halogen light source, and the reflected light from the test line was measured using a spectrometer.

First, the signal wavelength band, where the intensity changes depending on the amount of the antigen–antibody conjugate, was determined by spectrometric measurement of the labeled particles collected in the test line of the assay. Figure 1 shows the test fixture and the associated schematic. In the figure, the fiber-optic reflection probe (R400-7-UV-VIS, Ocean Optics, Dunedin, FL, USA) consists of six round fibers and a single core fiber bundle for illumination and reading, respectively. The white light from a halogen light source (HL-2000-LL, Ocean Optics, Dunedin, FL, USA) was used to irradiate the test line through the illumination fibers of the optical refection probe, and the intensity of the reflected light was measured using a spectrometer (USB2000 + UV–VIS, Ocean Optics, Dunedin, FL, USA).

### 2.2. Colorimetric Photometry of LFIA

**Figure 2:**
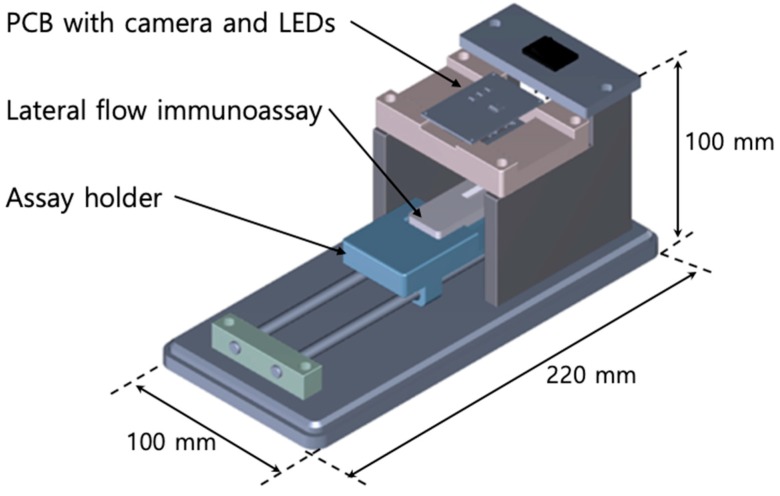
An assay reader for the quantitative analysis using a color camera. The assay reader consists of a camera, LEDs, a holder onto which the assay is mounted, and rails for transport. There is a cover for separation from ambient light, but it is not shown in the figure. PCB = printed circuit board.

The colorimetric photometry of the LFIA was performed using a custom-made reader device, as shown in Figure 2. After attaching the LFIA to the assay holder of the reader, it was manually pushed to the bottom of the printed circuit board (PCB). The color image of the test line was obtained through a camera module (STC-SC133USB-B, Sony ICX445AQ CCD installed, Sensor Technologies America, Inc., Carrollton, TX, USA) mounted on the PCB, as shown in Figure 2. The Bayer filter is a filter array located on the surface of a color image sensor. One pixel of the Bayer filter usually consists of one red, two green, and one blue grid to simulate the human eye which is more sensitive to green light. Each grid passes light in the red, green, and blue wavelength band. For the color image sensor (i.e., color CCD) used in this study, the center wavelengths of red, green, and blue light passing through the Bayer filter were 613 nm, 537 nm, and 456 nm, respectively, according to the manufacturer’s specifications. Strictly speaking, a color camera distorts the natural phenomena coming in to simulate the human eye. However, quantification of the assay in this study was performed using one of the three bands, and no more than two bands were mixed for measurements. The PCB in the reader included white LEDs for lighting around the test line, and the LEDs were powered on for a few seconds, including the moment the camera collected the image.

**Figure 3:**
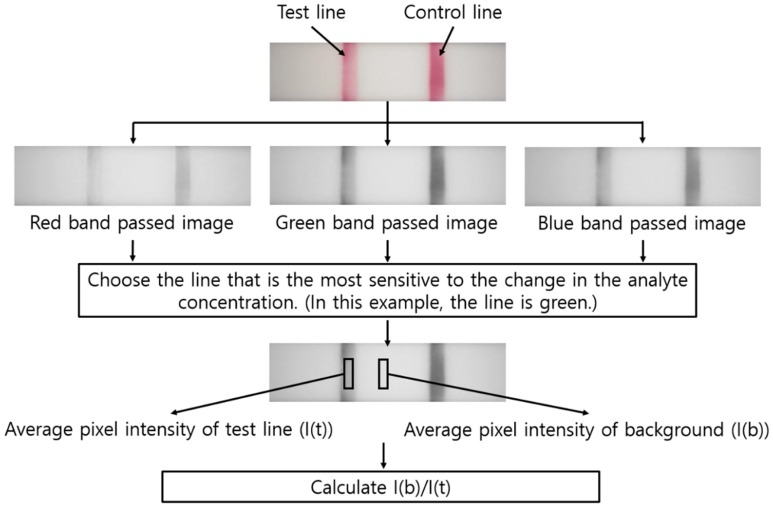
Analysis process of the color images for LFIA quantification. The control line captures any particle passing through, and thereby is used to ensure the proper sample flow in an LFIA. Only the test line area was analyzed.

The acquired image was stored in the memory card of the PCB. Then, the image was transferred from the memory card to the desktop computer and analyzed using customized MATLAB code, as shown in Figure 3. The image obtained from the color camera of the reader device is shown at the top of Figure 3. The image passed through the Bayer filter, and the pixel intensity was individually stored as three color bands (i.e., red, green, and blue). From the experiment in Figure 1, we selected the color band (i.e., red, green, or blue) in which the pixel intensity of the test line varied the most with respect to the change in the target material concentration in the assay. This selected color channel was used to quantify the assay. Let I(t) and I(b) be the average pixel intensity values of the region of interest in the test line portion and the background portion of the assay membrane, respectively. I(b)/I(t) was calculated for the test line that formed when the sample solution was developed on the assay. The SigmaPlot software (v. 12.5; Systat Software, Inc., San Jose, CA, USA) was used for statistical analysis (i.e., Bonferroni’s one way analysis of variance (ANOVA)) of photometry.

### 2.3. Assay and Sample Used to Verify the Readout Method

The assay used in this study was used to quantify creatine kinase–muscle/brain (CK-MB) in whole blood samples for the diagnosis of acute myocardial infarction (provided by Nano Advanced Bio, Seongnam-si, Korea) [12]. CK-MB is one of the enzymes found in cardiac muscle cells and the CK-MB test is commonly used for the clinical assessment of shortness of breath, cardiac risk, and acute myocardial infarction. The nitrocellulose porous membrane (HF135, Merck Millipore, Burlington, MA, USA) is pre-coated with capture reagent polyclonal CK-MB antibodies (G-113-C, BiosPacific, Emeryville, CA, USA) on the test line region of the assay. During testing, the sample reacts with a mouse IgG gold label (BGCMIG, Bore Da Biotech, Seongnam-si, Korea) coated with a monoclonal CK-MB antibody (10-C40A, Fitzgerald, Acton, MA, USA) at the conjugate release pad (8964, Ahlstrom, Helsinki, Finland) of the assay. After the sample conjugation, the mixture flows through the porous membrane by capillary action to react with capture reagent and generate a colored line, which is the aggregate of the labeled particles (i.e., gold particles). The presence of this colored test line indicates a positive result, and the absence of a test line indicates a negative result. Because blood samples were not available for this study, CK-MB (1-028, BioSpecifics, Lynbrook, NY, USA) was diluted with serum (22000, SeraCare Life Sciences, Milford, MA, USA) to obtain 0, 2, 4, 10, 50, and 100 ng/mL solutions that were used as samples. These concentrations reflect the criteria for clinical positive and negative determinations, where the concentration of CK-MB in the blood deemed negative for myocardial infarction is less than 1.9–8.7 ng/mL [13,14]. Before the spectrometry and photometry measurements using the assay, the prepared sample solution was injected through the sample inlet of the assay followed by 15 min of incubation for complete antigen–antibody reaction.

## 3. Results and Discussion

### 3.1. Optimum Wavelenghth Band for Assay Quantification

**Figure 4:**
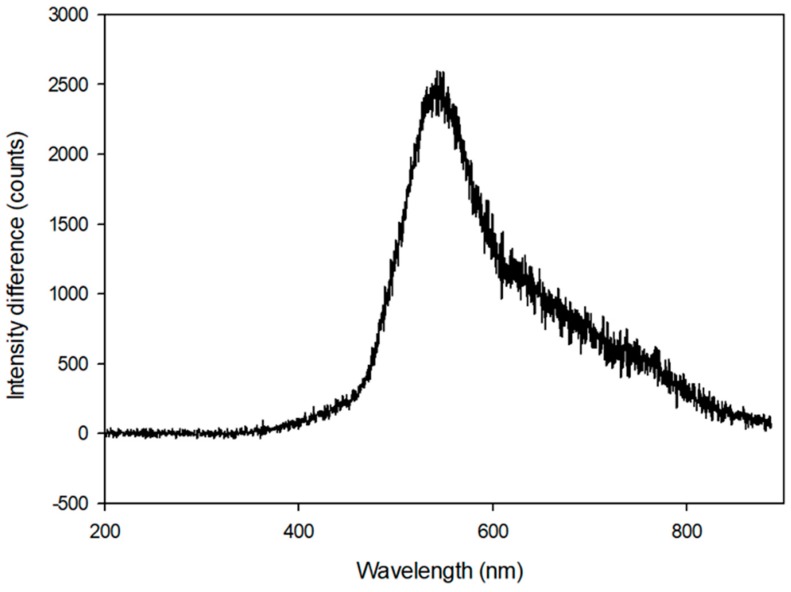
The spectrometric intensity difference between the light reflected from the test line (i.e., aggregate of gold labeled particles) area and the membrane background. The difference is greatest at a wavelength of 546 nm.

Spectrometry measurements for the LFIA were performed as shown in Figure 1. After the test line was formed by developing a sample (100 ng/mL CK-MB) in the assay, the spectrometric intensity difference between the reflected light of the test line area and the membrane background was calculated, and the result is shown in Figure 4. As shown in the figure, the color that formed when the labeled particles collected on the test line showed the greatest intensity change at a wavelength of 546 nm, which is around the center wavelength of the green channel in the Bayer filter used in the camera module of the reader (i.e., 537 nm).

**Figure 5:**
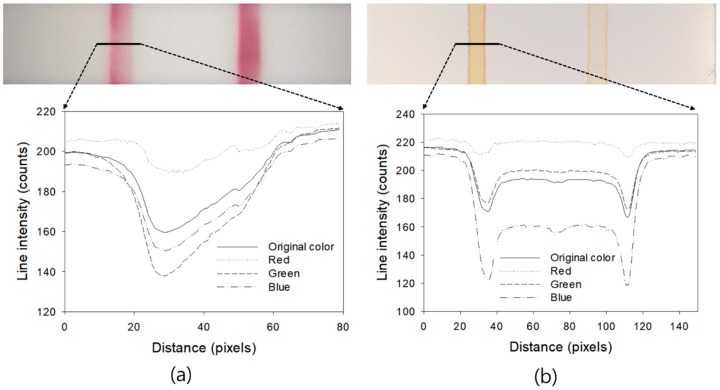
Comparison of the pixel intensity profiles of the original color, red, green, and blue images for an LFIA test line using gold nanoparticles (**a**) and iron oxide nanoparticles (**b**) as the labeling particles.

The color channel with the highest sensitivity depends on the color of the labeling particles used in the assay. In Figure 5, the top of (a) and (b) are the images of the test lines formed by developing a sample (100 ng/mL CK-MB) in the assay and simulating test lines by spraying iron oxide nanoparticles on a porous membrane, respectively. Iron oxide nanoparticles have been used in other LFIAs [6] and are brown in color. The graph below each image shows the pixel intensity profile for each color channel (i.e., original color, red, green, and blue) for the solid line portion of the assay image. As shown in Figure 5, the green color channel has the most sensitive change for the formation of the test line when using gold nanoparticles (a), which is similar to the experimental results shown in Figure 4. On the other hand, when using iron oxide nanoparticles (b), the blue channel is the most sensitive. In this case, the test line and the membrane background intensity difference measurement showed maximum values at 487 nm and the blue filter center wavelength of the image sensor used was 456 nm. Therefore, an assay analysis using a blue band image maximizes the efficiency of quantification when iron oxide nanoparticles, shown in Figure 5b, are used as the labeling particles in the assay. When the iron oxide solution was applied to the membrane, the particles were aggregated and formed a thick line at the edge of the test line, as shown in the test line image of Figure 5b. This is considered to be a specific phenomenon occurring at the interface between the liquid and the membrane when the iron oxide particle solution is dried after being sprayed. However, these lines were not formed in the case of Figure 5a, in which the test line was formed by the reaction of the actual antigen-antibody.

### 3.2. Quantitative Analysis of the Assay

As the concentration of the target material increases in the sample solution, a darker test line forms, and its I(t) value decreases. Assuming that I(b) remains the same at different concentrations, the value of I(b)/I(t) increases as the amount of the target substance increases. Such a measurement of the ratio of the test line and the background pixel intensity effectively compensates for the output drift due to the change of illumination, which may be different at each shooting time. This is because the degree of change in the pixel intensity is the same for the background and test lines and, therefore, can be canceled out in the ratio calculation.

**Figure 6:**
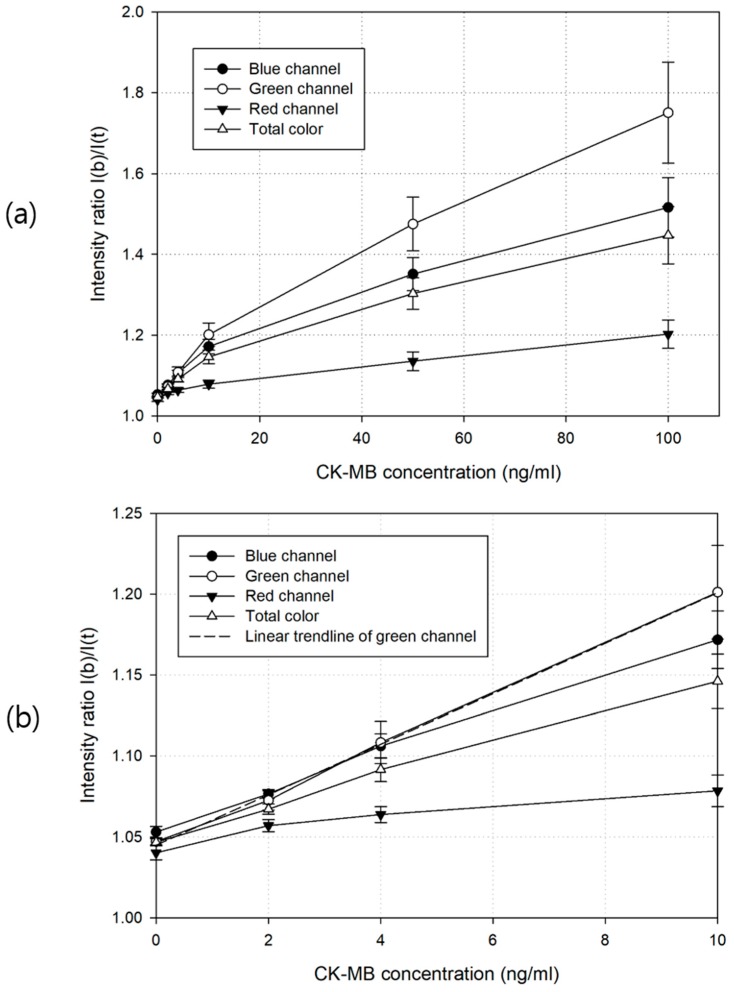
Pixel intensity response curves using the total color (i.e., original color), red, green, and blue images of the LFIA (**a**) and the enlarged response curves for the creatine kinase–muscle/brain (CK-MB) concentration range of 0 to 10 ng/mL (**b**). An average of 10 measurements is shown, and the error bars show the standard deviation. The dashed line in (**b**) shows the linear regression result of the green channel.

Figure 6 shows the results of the quantitative analysis of the assay using color images taken by the assay reader, as shown in Figure 2, and applying the image analysis algorithm introduced in Figure 3. The change in the intensity ratio with increasing concentration of the target substance (CK-MB) in the sample was the largest in the green channel and the smallest in the red channel, as can be inferred from the results in Figure 4. The linear range of the CK-MB concentration output curves in each color channel ranged from 0 to 10 ng/mL. In Figure 6b, the linear trendline of the green channel in this linear range was y = 1.0449 + 0.0156x and R^2^ = 0.9342, and the detection limit was 2 ng/mL. Because the cut-off concentration of CK-MB is 1.9 to 8.7 ng/mL, the detection limit of 2 ng/mL is appropriate for diagnosis. Bonferroni’s one-way ANOVA test was performed to investigate whether the quantification method using the green channel of the assay color image was statistically significant compared with the method using the other color channel and the method using the original color. The results are shown in Table 1. The red channel method showed the largest mean value difference at each CK-MB concentration compared with the original color image method. However, this is because the red channel method has the lowest intensity ratio as shown in Figure 6. The blue channel method had slightly larger mean values and a slightly smaller *p*-value than the green channel method at 2 ng/mL of CK-MB. When the concentration of CK-MB was higher than that, the method of using the green channel was the highest level of improvement in the measurement performance. The method using the blue channel did not show the statistically significant difference compared with the method using the original color image at 50 and 100 ng/mL CK-MB.

As shown in Figure 4, the intensity difference in reflected light between the test line area and the background area of the assay could be detected at wavelengths greater than 600 nm, including the red band. However, the light was filtered by an on-chip infrared cut-off filter, which is commonly used in most image sensors, and did not contribute to the assay quantification. The change in the intensity ratio according to the change in the analyte concentration in the red color channel was relatively small, as shown in Figure 6. In this figure, the total color refers to the original color image obtained from the camera (i.e., without color channel separation). In the case of the total color analysis, the degradation of quantification sensitivity occurred because the color information of the labeled particles was distributed near the wavelength of 546 nm, but the response spectrum of the image sensor was in the range of 400–700 nm. Within this range, noises, such as the background color of the porous membrane, the color of the liquid sample, and ambient light, may enter the image and cause interference. Even in the case of an analysis using a monochromic image sensor, which is currently used in most optical reader devices, degradation of the quantitative analysis efficiency cannot be avoided without a bandpass filter [10,11]. As shown in Figure 6, the degree of increased sensitivity became larger as the concentration of the target substance increased.

Wavelength or frequency domain analysis using an optical spectrometer, as shown in Figure 1, may be one way to improve assay quantification efficiency. However, this analysis will cause other problems, such as high cost and large size of the assay reader, which will hinder the realization of an LFIA system for point-of-care and rapid testing. Therefore, utilization of a color camera and a corresponding Bayer-filtered image for the quantitative assay reader in this research is expected to effectively solve the problems of previous methods with minimal instrumentation and a simple analysis process.

## 4. Conclusions

In this research, Bayer-filtered images obtained from a color camera were used to improve the performance of a quantitative LFIA system. It was demonstrated that the specific color component analysis method showed improved sensitivity for quantifying captured particles or the analyte concentration in an LFIA compared with that obtained utilizing traditional total color imaging. Comparisons with existing methods for quantitatively analyzing the same analyte, interference, and recovery analysis were not performed in this study, but these will be needed in the future. The reader system with a color camera offers many other potential advantages. It allows for a background adjustment when the test assay is used with a whole blood sample. Red blood cells frequently leave a red-colored background (i.e., noise) in an LFIA membrane and interfere with the intensity signal of the test line. If the test line signal is in the blue bandpass range, the blue color analysis method can effectively remove the noise in the red range, and the overall signal-to-noise ratio can be improved. The color separation method in this research can also be applied for luminescence- or fluorescence-based LFIA quantification. In this case, the color channel needs to be selected to maximize the sensitivity of the test line color and minimize the interference of the excitation light.

## Figures and Tables

**Table 1:** ANOVA test results of analysis methods using each color channel of the assay (vs. the method of analysis using original color image). If the *p* value is greater than 0.05, it means that there is no statistically significant difference in quantitative analysis performance. CK-MB = creatine kinase–muscle/brain.

CK-MB	Comparison	Difference of Means	*t*	*p*	*p* < 0.05
2 ng/mL	Original vs. Red	0.0102	5.562	<0.001	Yes
	Original vs. Green	0.0054	5.562	0.017	Yes
	Original vs. Blue	0.0094	5.124	<0.001	Yes
4 ng/mL	Original vs. Red	0.0278	7.114	<0.001	Yes
	Original vs. Green	0.0168	4.296	<0.001	Yes
	Original vs. Blue	0.0146	3.728	0.002	Yes
10 ng/mL	Original vs. Red	0.0677	7.744	<0.001	Yes
	Original vs. Green	0.0550	6.292	<0.001	Yes
	Original vs. Blue	0.0256	2.928	0.018	Yes
50 ng/mL	Original vs. Red	0.1720	8.509	<0.001	Yes
	Original vs. Green	0.1670	8.275	<0.001	Yes
	Original vs. Blue	0.0482	2.383	0.068	No
100 ng/mL	Original vs. Red	0.3030	8.2	<0.001	Yes
	Original vs. Green	0.2450	6.618	<0.001	Yes
	Original vs. Blue	0.0691	1.869	0.209	No
